# Fruit and vegetable intake and prostate cancer risk in the European Prospective Investigation into Cancer and Nutrition (EPIC)

**DOI:** 10.1002/ijc.30741

**Published:** 2017-05-15

**Authors:** Aurora Perez‐Cornago, Ruth C. Travis, Paul N. Appleby, Konstantinos K. Tsilidis, Anne Tjønneland, Anja Olsen, Kim Overvad, Verena Katzke, Tilman Kühn, Antonia Trichopoulou, Eleni Peppa, Maria Kritikou, Sabina Sieri, Domenico Palli, Carlotta Sacerdote, Rosario Tumino, H. B(as) Bueno‐de‐Mesquita, Antonio Agudo, Nerea Larrañaga, Elena Molina‐Portillo, Eva Ardanaz, Maria‐Dolores Chirlaque, Cristina Lasheras, Pär Stattin, Maria Wennberg, Isabel Drake, Johan Malm, Julie A. Schmidt, Kay‐Tee Khaw, Marc Gunter, Heinz Freisling, Inge Huybrechts, Dagfinn Aune, Amanda J Cross, Elio Riboli, Timothy J. Key

**Affiliations:** ^1^ Cancer Epidemiology Unit, Nuffield Department of Population HealthUniversity of OxfordOxfordUnited Kingdom; ^2^ Department of Hygiene and EpidemiologyUniversity of Ioannina School of MedicineIoanninaGreece; ^3^ Department of Epidemiology and Biostatistics, School of Public HealthImperial College LondonLondonUnited Kingdom; ^4^ Danish Cancer Society Research CenterCopenhagenDenmark; ^5^ Department of Public Health, Section for EpidemiologyAarhus UniversityAarhus CDenmark; ^6^ Division of Cancer EpidemiologyGerman Cancer Research Center (DKFZ)HeidelbergGermany; ^7^ Hellenic Health FoundationAthensGreece; ^8^ WHO Collaborating Center for Nutrition and Health, Unit of Nutritional Epidemiology and Nutrition in Public Health, Dept. of Hygiene, Epidemiology and Medical StatisticsUniversity of Athens Medical SchoolAthensGreece; ^9^ Epidemiology and Prevention UnitFondazione IRCCS Istituto Nazionale dei TumoriMilanoItaly; ^10^ Cancer Risk Factors and Life‐Style Epidemiology UnitCancer Research and Prevention Institute—ISPOFlorenceItaly; ^11^ Unit of Cancer Epidemiology, AO Citta' della Salute e della Scienza‐University of Turin and Center for Cancer Prevention (CPO‐Piemonte)TurinItaly; ^12^ Cancer Registry and Histopathology Unit “Civic—M.P. Arezzo “Hospital ASP RagusaItaly; ^13^ Department for Determinants of Chronic Diseases (DCD)National Institute for Public Health and the Environment (RIVM)BilthovenThe Netherlands; ^14^ Department of Social and Preventive Medicine, Faculty of MedicineUniversity of MalayaKuala LumpurMalaysia; ^15^ Unit of Nutrition and Cancer, Cancer Epidemiology Research Program, Catalan Institute of Oncology‐IDIBELL, L'Hospitalet de LlobregatBarcelonaSpain; ^16^ Public Health Division of GipuzkoaRegional Government of the Basque CountrySpain; ^17^ CIBER of Epidemiology and Public Health (CIBERESP)MadridSpain; ^18^ Escuela Andaluza de Salud Pública, Instituto de Investigación Biosanitaria ibs, GRANADAHospitales Universitarios de Granada/Universidad de GranadaGranadaSpain; ^19^ Navarra Public Health InstitutePamplonaSpain; ^20^ Navarra Institute for Health Research (IdiSNA) PamplonaPamplonaSpain; ^21^ Department of EpidemiologyRegional Health Council, IMIB‐ArrixacaMurciaSpain; ^22^ Department of Health and Social SciencesUniversidad de MurciaMurciaSpain; ^23^ Department of Functional Biology, Faculty of MedicineUniversity of OviedoAsturiasSpain; ^24^ Department of Surgical SciencesUppsala UniversityUppsalaSweden; ^25^ Department of Surgical and Perioperative Sciences, Urology and AndrologyUmea UniversityUmeaSweden; ^26^ Department of Public Health and Clinical MedicineNutritional Research, Umeå UniversityUmeåSweden; ^27^ Diabetes and Cardiovascular Disease—Genetic Epidemiology; Department of Clinical Sciences in MalmöLund UniversityLundSweden; ^28^ Department of Translational Medicine Clinical ChemistryLund University; Skåne University HospitalMalmöSweden; ^29^ University of Cambridge School of Clinical MedicineCambridgeUnited Kingdom; ^30^ Section of Nutrition and MetabolismInternational Agency for Research on CancerLyonFrance

**Keywords:** prostate cancer, fruit, vegetable, tumor subtypes, prospective

## Abstract

Several dietary factors have been studied in relation to prostate cancer; however, most studies have not reported on subtypes of fruit and vegetables or tumor characteristics, and results obtained so far are inconclusive. This study aimed to examine the prospective association of total and subtypes of fruit and vegetable intake with the incidence of prostate cancer overall, by grade and stage of disease, and prostate cancer death. Lifestyle information for 142,239 men participating in the European Prospective Investigation into Cancer and Nutrition from 8 European countries was collected at baseline. Multivariable Cox regression models were used to estimate hazard ratios (HRs) and 95% confidence intervals (CIs). After an average follow‐up time of 13.9 years, 7,036 prostate cancer cases were identified. Compared with the lowest fifth, those in the highest fifth of total fruit intake had a significantly reduced prostate cancer risk (HR = 0.91; 95% CI = 0.83–0.99; *p*‐trend = 0.01). No associations between fruit subtypes and prostate cancer risk were observed, except for citrus fruits, where a significant trend was found (HR = 0.94; 95% CI = 0.86–1.02; *p*‐trend = 0.01). No associations between total and subtypes of vegetables and prostate cancer risk were observed. We found no evidence of heterogeneity in these associations by tumor grade and stage, with the exception of significant heterogeneity by tumor grade (*p*_heterogeneity_<0.001) for leafy vegetables. No significant associations with prostate cancer death were observed. The main finding of this prospective study was that a higher fruit intake was associated with a small reduction in prostate cancer risk. Whether this association is causal remains unclear.

## Abstract 2

**What's new?**

The role of diet in prostate‐cancer etiology is uncertain, and associations may vary by tumor characteristics. In this prospective, longitudinal study, the authors examined the association of total and subtypes of fruit and vegetable intake with the overall incidence of prostate cancer. They then analyzed incidence by grade, stage of disease, and prostate‐cancer death. They found that higher fruit intake was associated with a small reduction in prostate cancer risk, and that this association did not differ by tumor characteristics.

**Abbreviations**
BMI: body mass index
CIs: confidence intervals
EPIC: European Prospective Investigation into Cancer and Nutrition
FFQ: food‐frequency questionnaire
ICD: International Statistical Classification of Diseases, Injuries and Causes of Death
HRs: hazard ratios
ORs: odds ratios
PCa: prostate cancer
PSA: prostate‐specific antigen
TNM: tumor‐node‐metastasis
UK: United Kingdom
WCRF/AICR: World Cancer Research Fund/American Institute for Cancer Research

Prostate cancer is the second most frequently diagnosed cancer in men worldwide.^1^ Due to the wide international variation in prostate cancer incidence, environmental and lifestyle factors, such as dietary factors, have been proposed as possible risk factors for the disease.^2^ However, the role of diet in the development of prostate cancer is uncertain, and associations may vary depending on the grade or stage of the disease.^3^

Several dietary factors have been studied in relation to prostate cancer, among them fruit and vegetable intake,^4^ but studies vary in design, most of them do not report results subdivided by grade and stage of prostate cancer or fruit and vegetable subtypes, and the results obtained so far are inconclusive.^4^, ^5^, ^6^, ^7^, ^8^, ^9^ High fruit and vegetable intake may be associated with reduced deoxyribonucleic acid (DNA) oxidation, cell damage, and low‐grade inflammation, as well as increasing the activity of detoxification enzymes.^10^ Furthermore, different types of fruit and vegetables consumed may provide different bioactive compounds with different actions on prostate cancer.^4^, ^11^ In recent years, special attention has been paid to the possible protective effect of the consumption of cruciferous vegetables and tomatoes on prostate cancer, although to date results are not conclusive.^12^, ^13^ Regarding fruit consumption, most prospective studies have found no association with prostate cancer development.^4^, ^5^, ^6^, ^9^ The latest meta‐analysis from the World Cancer Research Fund/American Institute for Cancer Research (WCRF/AICR) stated that no conclusion could be reached on whether fruit and vegetable consumption is associated with prostate cancer risk^3^; this meta‐analysis did not differentiate between grade and stage of the disease (stage and grade are grouped together as advanced/high grade or non‐advanced/low grade), and only included a limited number of fruit and vegetable subtypes (namely cruciferous vegetables and tomatoes); therefore, more studies are needed.

The aim of this study was to examine the association of fruit and vegetable consumption with prostate cancer risk in the European Prospective Investigation into Cancer and Nutrition (EPIC). In this cohort, based on analyses of the first 1,104 cases, it was previously reported that total consumption of fruits and vegetables was not related to the development of total prostate cancer.^14^ However, there were insufficient data to investigate whether the association varied by tumor characteristics, whether there was an association with prostate cancer death, or to investigate the associations with different fruit and vegetable subtypes. Therefore, our aim is to report results from an extension of this earlier work, including now 7,036 prostate cancer cases, as well as to analyze the main fruit and vegetable subtypes, and to describe whether any associations differ by tumor grade or stage, and prostate cancer death.

## Material and Methods

### Subjects and study design

EPIC is an ongoing multicenter, prospective cohort study investigating the relationships of dietary and lifestyle factors with cancer and other chronic diseases carried out in 23 centers in 10 European countries: Denmark, France, Germany, Greece, Italy, The Netherlands, Norway, Spain, Sweden and the United Kingdom (UK). Participants were mostly recruited from the general population. The full cohort comprises 519,978 participants including 153,457 men, most aged 35–70 years, recruited between 1992 and 2000. The details of the study design used in the EPIC study have been described elsewhere.^15^

A study flowchart is shown in Supporting Information Figure 1. Since only women were recruited in France, Norway, Naples (Italy) and Utrecht (The Netherlands), these centers where not included in the current study, and data from 19 centers in 8 countries were included in this analysis. A total of 367,898 women were excluded. We also excluded men who were diagnosed with cancer (except non‐melanoma skin cancer) before recruitment (*n* = 3,972), those with missing dates of prostate cancer diagnosis (*n* = 14) or follow‐up (*n* = 1,433), those aged <20 years at recruitment (*n* = 2), as well as those who had no non‐dietary or dietary data, or men with an extreme energy intake in relation to estimated requirement (*n* = 5,766).^16^ Finally, a total of 142,239 men were available for analysis.

### Assessment of dietary intake and other predictor variables

At baseline, information was collected on lifestyle, health status, socio‐demographic characteristics, anthropometry and medical history.^15^ Information on dietary intake during the year before recruitment was collected by country‐ or center‐specific validated dietary questionnaires. Most centers used self‐administered dietary questionnaires, however, in Greece, Ragusa (Italy), and Spain participants were interviewed by trained staff members. In Malmö (Sweden) dietary intake was assessed using a modified diet history method which combined information from a self‐administered diet questionnaire, a 7‐day food registration and a 1‐hr interview. With the purpose of improving the comparability of dietary data across the participating centers, dietary intakes from the questionnaires were calibrated using a standardized, computer‐based, 24‐hr dietary recall method in an 8% random sample of the whole EPIC cohort. Information on validation of the dietary questionnaires has been published previously.^17^

On the basis of this information, daily fruit (fresh fruit only) and vegetable (potatoes and dried beans not included) consumption was based on the food group classification previously used for the EPIC cohort.^18^ In this study, fruit and vegetables were in turn classified into sub‐groups. The subtypes of fruit considered were citrus fruits (*e.g*., oranges, lemons), apples and pears, and bananas. For vegetables, the following sub‐groups were used: cruciferous vegetables (*e.g*., broccoli, cabbage), leafy vegetables (*e.g*., spinach, lettuce), fruiting vegetables (*e.g*., tomato, sweet pepper, eggplant), tomatoes (raw, cooked, sauce) and root vegetables (*e.g*., carrot, beetroot).

### Endpoints

The main source of information on prostate cancer cases was population‐based cancer registries. In Germany and Greece follow‐up was based on a combination of methods, including health insurance records, cancer and pathology registries, as well as active follow‐up through participants or relatives; self‐reported incident cancers were verified through medical records. Follow‐up began at the date of recruitment and was censored at the date of last known contact, or at the date of diagnosis of cancer, death, emigration or the end of the follow‐up period, whichever came first. A total of 7,036 men developed prostate cancer (code: C61) according to the 10th Revision of the International Statistical Classification of Diseases, Injuries and Causes of Death (ICD).^19^

Information on stage [tumor‐node‐metastasis (TNM) staging code] and grade of prostate cancer (based on Gleason sum) was collected from each center, where possible. Grade was stratified as low‐intermediate (Gleason score of < 8, or grade coded as well, moderately, or poorly differentiated; *n* = 3,757) or high (Gleason score of ≥ 8, or grade coded as undifferentiated; *n* = 726) grade. Localized stage included those confined within the prostate and with no metastases at diagnosis (TNM staging score of ≤ T_2_ and N_0_/N_x_ and M_0_, or stage coded in the recruitment center as localized; *n* = 2,641). Advanced cases included tumors that had spread beyond the prostate at diagnosis (T_3_‐T_4_ and/or N_1_‐N_3_ and/or M_1_, and/or stage coded in the recruitment center as metastatic; *n* = 1,389). Fatal cases were those who died of prostate cancer (*n* = 936).

### Statistical analysis

Hazard ratios (HRs) and 95% confidence intervals (CIs) were estimated using Cox proportional hazards models using age as the underlying time variable. The date of last follow‐up ranged from January 2011 in Germany to October 2013 in Spain. All analyses were stratified by center and age (<50, 50–54.9, 55–59.9, 60–64.9, 65–69.9 and ≥70 years) at recruitment. To check for violation of the proportional hazards assumption we used time‐varying covariates and Schoenfeld residuals, which indicated no evidence of deviation from the proportional hazards assumption. Fruit and vegetable intakes (g/day) were divided into fifths based on the distribution in the EPIC cohort and also modeled as continuous variables in increments of 100 g/day (approximately equivalent to one portion). Tests for linear trend were performed using a pseudo‐continuous variable equal to the median value in each fifth of intake divided by 100. All models were adjusted for educational level (no degree or equivalent, degree or equivalent, unknown), smoking status (never, former, current, unknown), marital status (married or cohabiting, not married or cohabiting, unknown), diabetes (no, yes, unknown), physical activity (inactive, moderately inactive, moderately active, active, unknown^20^), height (<170, 170–174, 175–179, ≥ 180 cm, unknown), BMI (<22.5, 22.5–24.9, 25–29.9, ≥ 30 kg/m^2^, unknown), and total energy intake (fifths). Participants with missing values were assigned an “unknown” category; <3% of values were missing for each covariate, with the exception of marital status, for which 30% of values were missing.

Tests for heterogeneity of trends for case‐defined characteristics [histologic grade (low‐intermediate or high), tumor stage (localized or advanced), age at diagnosis (<65 years, ≥65 years), and time between blood collection and diagnosis (<5 years, ≥5 years)] were performed. For this, we fitted stratified Cox models based on competing risks and compared the risk coefficients and standard errors in the subgroups of interest after excluding cases of unknown grade or stage.^21^ For the non‐case‐defined factors [age at recruitment (<65 years, ≥65 years), BMI (<25 kg/m^2^, ≥25 kg/m^2^), and country (Denmark, Germany, Greece, Italy, The Netherlands, Spain, Sweden, UK)], the test for heterogeneity was assessed by using likelihood ratio tests to compare the Cox models with and without interaction terms for the dietary variable and the relevant factor. All analyses were performed using Stata version 14.1 (Stata Corporation, College Station, TX), all tests of significance were two‐sided, and a *p* values <0.05 was considered statistically significant.

## Results

After an average of 13.9 years of follow‐up, 7,036 men were diagnosed with prostate cancer among the 142,239 participants included in this study. The median age at prostate cancer diagnosis was 68 years (range, 41–95 years). Baseline details of participants are shown in Table 1. Some characteristics varied by fruit and vegetable consumption. For example, men in the highest fifths of fruit and vegetable intake were older at recruitment and at diagnosis, less likely to smoke, more likely be diabetic, were shorter and with a higher BMI and energy intake.

**Table 1:** Baseline characteristics of 142,239 men in EPIC (1992–2013) according to observed fruit and vegetable intake

	Fifths of observed fruit intake			Fifths of observed vegetable intake		
	1	3	5	1	3	5
No. of men	28,447	28,446	28,447	28,448	28,447	28,447
Age at recruitment^a^, y	50.7 (10.0)	52.2 (10.2)	51.6 (10.1)	50.6 (10.1)	51.9 (9.7)	52.2 (11.2)
Age at diagnosis^a^, y	67.0 (6.6)	68.4 (6.6)	68.0 (6.8)	67.4 (6.6)	67.6 (6.5)	68.8 (7.3)
Smoking, *n* (%)						
Never	7,848 (27.6)	9,951 (35.0)	9,683 (34.0)	9,958 (35.0)	9,403 (33.1)	8,860 (31.1)
Former	8,903 (31.3)	10,713 (37.7)	10,669 (37.5)	8,992 (31.6)	10,742 (37.8)	10,491 (36.9)
Current	11,445 (40.2)	7,430 (26.1)	7,507 (26.4)	9,225 (32.4)	8,012 (28.2)	8,302 (29.2)
Educational level, *n* (%)						
No degree	20,914 (73.5)	19,359 (68.1)	21,264 (74.7)	22,516 (79.1)	19,310 (67.9)	19,886 (69.9)
Degree	6,872 (24.2)	8,164 (28.7)	6,464 (22.7)	5,667 (19.9)	8,396 (29.5)	7,348 (25.8)
Physical activity, *n* (%)						
Inactive	5,660 (19.9)	5,122 (18.0)	5,461 (19.2)	5,510 (19.4)	4,640 (16.3)	6,736 (23.7)
Moderately inactive	8,835 (31.1)	8,714 (30.6)	8,152 (28.7)	9,138 (32.1)	8,822 (31.0)	8,053 (28.3)
Moderately active	6,678 (23.5)	6,837 (24.0)	7,045 (24.8)	7,006 (24.6)	6,712 (23.6)	7,251 (25.5)
Active	6,561 (23.1)	7,028 (24.7)	7,464 (26.2)	6,297 (22.1)	7,426 (26.1)	6,164 (21.7)
Diabetes at baseline, *n* (%)						
No	27,015 (95.0)	26,682 (93.8)	26,834 (94.3)	27,106 (95.3)	26,898 (94.6)	26,408 (92.8)
Yes	736 (2.6)	1,030 (3.6)	1,188 (4.2)	715 (2.5)	881 (3.1)	1,614 (5.7)
Marital status, *n* (%)						
Married	13,613 (47.9)	16,124 (56.7)	16,114 (56.6)	16,853 (59.2)	15,428 (54.2)	16,609 (58.4)
Not married	4,536 (15.9)	3,719 (13.1)	2,719 (9.6)	5,064 (17.8)	3,666 (12.9)	2,635 (9.3)
Height^a^, cm	175.7 (7.1)	175.3 (7.2)	172.5 (7.4)	175.5 (7.2)	175.3 (7.2)	172.6 (7.5)
BMI^a^, kg/m^2^	26.2 (3.7)	26.3 (3.6)	27.1 (3.7)	26.2 (3.6)	26.3 (3.6)	27.2 (3.8)
Total energy intake^a^, Kcal/d	2,260 (643)	2,398 (638)	2,614 (687)	2,208 (636)	2,450 (644)	2,533 (689)

Percentages do not sum to 100% due to missing data.

a Values are means (SD).

The estimated HRs for total prostate cancer risk across fifths of fruit intake, overall and for specific types of fruit are shown in Table 2. The only statistically significant association was observed for total fruit intake: compared with men in the lowest fifth of total fruit, the men in the highest fifth of total fruit intake had a HR 0.91 (95% CI: 0.83–0.99; *p*‐trend = 0.01). There was also some evidence of a significant trend for citrus fruits (HR in highest fifth = 0.94; 0.86–1.02; *p*‐trend = 0.01) although the risk in the highest fifth of intake was not statistically significant. No associations with total prostate cancer risk were observed for other subtypes of fruits (apples and pears, and bananas).

**Table 2:** Multivariable‐adjusted hazard ratios (95% CI) for total prostate cancer by fifths of observed fruit intake in 142,239 men in EPIC (1992–2013)

	Fifths of observed fruit intake					
	1	2	3	4	5	*p*‐trend^b^
Total fruit, g/day	≤ 66.7	>66.7 to ≤ 123.6	>123.6 to ≤ 197.3	>197.3 to ≤ 320.0	>320.0	
Cases, *n*	1,420	1,540	1,556	1,419	1,101	
HR (95% CI)	1 ref	1.04 (0.97–1.12)	1.02 (0.95–1.10)	1.01 (0.94–1.09)	0.93 (0.86–1.02)	0.04
Adjusted HR (95% CI)^a^	1 ref	1.03 (0.95–1.10)	1.00 (0.93–1.08)	0.99 (0.91–1.07)	0.91 (0.83–0.99)	0.01
Citrus fruit, g/day	≤ 5.8	>5.8 to ≤ 14.3	>14.3 to ≤ 36.8	>36.8 to ≤ 78.4	>78.4	
Cases, *n*	1,551	1,494	1,528	1,341	1,122	
HR (95% CI)	1 ref	1.08 (1.00–1.16)	1.04 (0.97–1.12)	0.96 (0.90–1.04)	0.96 (0.88–1.04)	0.03
Adjusted HR (95% CI)^a^	1 ref	1.06 (0.99–1.14)	1.02 (0.95–1.10)	0.95 (0.88–1.02)	0.94 (0.86–1.02)	0.01
Apple/pear, g/day	≤ 9.0	>9.0 to ≤ 28.0	>28.0 to ≤ 62.0	>62.0 to ≤ 116.4	>116.4	
Cases, *n*	1,238	1,465	1,383	1,436	1,514	
HR (95% CI)	1 ref	1.09 (1.01–1.18)	1.04 (0.96–1.13)	1.07 (0.99–1.17)	1.04 (0.96–1.13)	0.9
Adjusted HR (95% CI)^a^	1 ref	1.08 (1.00–1.17)	1.03 (0.95–1.12)	1.06 (0.97–1.15)	1.02 (0.94–1.11)	0.7
Banana, g/day	≤ 0.6	>0.6 to ≤ 7.1	>7.1 to ≤ 15.0	>15.0 to ≤ 43.0	>43.0	
Cases, *n*	1,270	1,357	1,430	1,496	1,483	
HR (95% CI)	1 ref	1.10 (1.01–1.20)	1.09 (1.01–1.19)	1.10 (1.01–1.19)	1.07 (0.99–1.17)	0.8
Adjusted HR (95% CI)^a^	1 ref	1.09 (1.00–1.19)	1.08 (0.99–1.17)	1.08 (0.99–1.17)	1.05 (0.96–1.14)	0.8

Cox regression analysis. All models are adjusted for age (underlying time variable) and stratified by recruitment center and age at recruitment.

a Additionally adjusted for educational level (no degree, degree, unknown), smoking status (never, former, current, unknown), marital status (married, not married, unknown), diabetes (yes, no, unknown), physical activity (inactive, moderately inactive, moderately active, active, unknown), height (<170, 170–174, 175–179, ≥ 180 cm, unknown), body mass index (<22.5, 22.5–24.9, 25–29.9, ≥ 30 kg/m^2^, unknown), and total energy intake (fifths).

b *p*‐values for trend were obtained using a pseudo‐continuous variable equal to the median value in each fifth of intake.

No association was found between total prostate cancer risk and vegetable intake, overall (HR = 1.02, 0.93–1.12) or by subtypes of vegetables (cruciferous vegetables, leafy vegetables, fruiting vegetables, tomatoes, root vegetables; Table 3).

**Table 3:** Multivariable‐adjusted hazard ratios (95% CI) for prostate cancer by fifth of observed vegetable intake in 142,239 men in EPIC (1992–2013)

	Fifths of observed vegetable intake					
	1	2	3	4	5	*p*‐trend^b^
Total vegetables, g/day	≤ 82.8	>82.8 to ≤ 126.4	>126.4 to ≤ 182.1	>182.1 to ≤ 281.6	>281.7	
Cases, *n*	1,526	1,451	1,457	1,549	1,053	
HR (95% CI)	1 ref	1.04 (0.96–1.12)	1.01 (0.93–1.09)	1.07 (0.99–1.16)	1.04 (0.95–1.14)	0.4
Adjusted HR (95% CI)^a^	1 ref	1.03 (0.96–1.11)	0.99 (0.92–1.07)	1.05 (0.97–1.14)	1.02 (0.93–1.12)	0.6
Cruciferous vegetables, g/day	≤ 3.0	>3.0 to ≤ 9.2	>9.2 to ≤ 18.7	>18.7 to ≤ 36.6	>36.6	
Cases, *n*	1,457	1,287	1,253	1,190	1,207	
HR (95% CI)	1 ref	1.06 (0.97–1.15)	1.05 (0.96–1.15)	1.07 (0.98–1.16)	1.06 (0.96–1.18)	0.4
Adjusted HR (95% CI)^a^	1 ref	1.06 (0.97–1.15)	1.04 (0.95–1.13)	1.05 (0.96–1.15)	1.06 (0.96–1.17)	0.5
Leafy vegetables, g/day	≤ 1.6	>1.6 to ≤ 6.0	>6.0 to ≤ 15.1	>15.1 to ≤ 36.9	>36.9	
Cases, *n*	1,773	1,453	1,218	1,034	916	
HR (95% CI)	1 ref	1.03 (0.96–1.11)	1.09 (1.00–1.18)	1.04 (0.95–1.14)	1.09 (0.97–1.22)	0.3
Adjusted HR (95% CI)^a^	1 ref	1.01 (0.94–1.09)	1.06 (0.97–1.16)	1.01 (0.92–1.11)	1.06 (0.95–1.19)	0.4
Fruiting vegetables, g/day	≤ 21.6	>21.6 to ≤ 36.5	>36.5 to ≤ 56.3	>56.3 to ≤ 97.8	>97.8	
Cases, *n*	1,642	1,590	1,465	1,365	974	
HR (95% CI)	1 ref	1.07 (1.00–1.15)	1.05 (0.97–1.13)	1.03 (0.95–1.11)	1.04 (0.95–1.14)	0.8
Adjusted HR (95% CI)^a^	1 ref	1.06 (0.99–1.14)	1.04 (0.96–1.12)	1.01 (0.94–1.10)	1.03 (0.94–1.13)	0.9
Tomatoes, g/day	≤ 9.0	>9.0 to ≤ 18.9	>18.9 to ≤ 30.8	>30.8 to ≤ 67.3	>67.3	
Cases, *n*	1,612	1,540	1,443	1,480	961	
HR (95% CI)	1 ref	1.05 (0.97–1.13)	1.04 (0.96–1.12)	1.01 (0.93–1.09)	1.09 (0.98–1.21)	0.2
Adjusted HR (95% CI)^a^	1 ref	1.04 (0.97–1.12)	1.03 (0.95–1.11)	1.00 (0.92–1.08)	1.08 (0.97–1.20)	0.2
Root vegetables, g/day	≤ 3.8	>3.8 to ≤ 8.9	>8.9 to ≤ 16.3	>16.3 to ≤ 35.5	>35.5	
Cases, *n*	1,416	1,371	1,353	1,334	1,562	
HR (95% CI)	1 ref	1.01 (0.93–1.09)	1.02 (0.94–1.10)	1.01 (0.93–1.09)	1.04 (0.96–1.13)	0.3
Adjusted HR (95% CI)^a^	1 ref	1.00 (0.92–1.08)	1.00 (0.92–1.08)	0.99 (0.92–1.07)	1.02 (0.94–1.11)	0.5

Cox regression analysis. All models are adjusted for age (underlying time variable) and stratified by recruitment center and age at recruitment.

a Additionally adjusted for educational level (no degree, degree, unknown), smoking status (never, former, current, unknown), marital status (married, not married, unknown), diabetes (yes, no, unknown), physical activity (inactive, moderately inactive, moderately active, active, unknown), height (<170, 170–174, 175–179, ≥ 180 cm, unknown), body mass index (<22.5, 22.5–24.9, 25–29.9, ≥ 30 kg/m^2^, unknown), and total energy intake (fifths).

b *p*‐values for trend were obtained using a pseudo‐continuous variable equal to the median value in each fifth of intake.

The associations of fruit and vegetable intake with risk for total prostate cancer, prostate cancer subdivided by grade and stage of disease, and for prostate cancer death, using both the observed and calibrated intakes, are shown in Tables 4 and 5. Results for observed and calibrated intake were similar in direction. There was a weak significant association between observed and calibrated total fruit intake and risk of total prostate cancer; an increase of 100 g/day was associated with lower risk of total prostate cancer [3% lower risk (95% CI = 0.95–0.99) for observed and 4% (95% CI = 0.94–0.99) lower risk for calibrated intake]. An increase of 100 g/day in citrus fruit intake was related to lower total prostate cancer risk (HR = 0.92, 0.86–0.98 for observed and HR = 0.88, 0.80–0.97 for calibrated intake; *p*‐trend = 0.01 and 0.009, respectively). There was no evidence of heterogeneity in separate analyses by grade and stage, with the exception of significant heterogeneity in the association by tumor grade for leafy vegetables for both observed intake (*p*_heterogeneity_ < 0.001; HR = 0.94, 0.76–1.17 for low‐intermediate grade and HR = 2.66, 1.54–4.58 for high grade cancer) and calibrated intake (*p*_heterogeneity_ < 0.001; HR = 0.97, 0.61–1.55 for low‐intermediate grade and HR = 8.98, 2.79–28.97 for high grade cancer). We observed no associations between fruit or vegetable intake (both overall and for specific subtypes) and prostate cancer death.

**Table 4:** Multivariable‐adjusted hazard ratios (95% CI) for prostate cancer per unit increase (per 100 g/day) of fruit intake in 142,239 men in EPIC (1992–2013)

		Observed intake			Calibrated intake		
	No. of cases	HR (95% CI)^a^	*P*‐trend^b^	*P* for het.^c^	HR (95% CI)^a^	*P*‐trend^b^	*P* for het.^c^
Total fruit							
Total PCa	7,036	0.97 (0.95–0.99)	0.01		0.96 (0.94–0.99)	0.006	
Grade							
Low‐intermediate	3,757	0.98 (0.96–1.01)	0.3		0.98 (0.94–1.01)	0.2	
High	726	0.98 (0.92–1.04)	0.4	0.8	0.97 (0.90–1.05)	0.4	0.8
Stage							
Localized	2,641	0.96 (0.93–0.99)	0.02		0.95 (0.91–0.99)	0.02	
Advanced	1,389	0.99 (0.95–1.04)	0.7	0.3	0.99 (0.93–1.04)	0.6	0.3
PCa death	936	0.98 (0.92–1.03)	0.4		0.97 (0.91–1.04)	0.5	
Citrus fruit							
Total PCa	7,036	0.92 (0.86–0.98)	0.01		0.88 (0.80–0.97)	0.009	
Grade							
Low‐intermediate	3,757	0.90 (0.82–0.99)	0.03		0.85 (0.75–0.97)	0.02	
High	726	0.89 (0.77–1.15)	0.6	0.7	0.93 (0.70–1.25)	0.6	0.6
Stage							
Localized	2,641	0.90 (0.81–1.00)	0.05		0.84 (0.72–0.98)	0.03	
Advanced	1,389	0.90 (0.77–1.05)	0.2	0.9	0.86 (0.69–1.08)	0.2	0.9
PCa death	936	0.92 (0.77–1.11)	0.4		0.89 (0.69–1.16)	0.4	
Apple/pear							
Total PCa	7,036	0.99 (0.95–1.03)	0.7		0.99 (0.93–1.04)	0.6	
Grade							
Low‐intermediate	3,757	1.01 (0.95–1.07)	0.8		1.01 (0.93–1.08)	0.9	
High	726	1.00 (0.88–1.14)	0.9	0.9	1.00 (0.85–1.17)	0.9	0.9
Stage							
Localized	2,641	0.98 (0.90–1.05)	0.5		0.98 (0.89–1.07)	0.6	
Advanced	1,389	1.04 (0.95–1.15)	0.4	0.3	1.04 (0.92–1.17)	0.5	0.4
PCa death	936	0.94 (0.83–1.05)	0.3		0.93 (0.81–1.07)	0.3	
Banana							
Total PCa	7,036	0.99 (0.91–1.08)	0.8		1.01 (0.85–1.19)	0.9	
Grade							
Low‐intermediate	3,757	0.97 (0.86–1.10)	0.6		0.99 (0.78–1.26)	0.9	
High	726	1.06 (0.81–1.38)	0.7	0.6	1.13 (0.67–1.90)	0.6	0.6
Stage							
Localized	2,641	0.95 (0.82–1.10)	0.5		0.94 (0.71–1.25)	0.7	
Advanced	1,389	0.92 (0.76–1.12)	0.4	0.8	0.90 (0.62–1.32)	0.6	0.8
PCa death	936	1.07 (0.86–1.34)	0.5		1.16 (0.75–1.80)	0.5	

PCa: prostate cancer.

Cox regression analysis. All models are stratified by center and age at recruitment and adjusted for age (underlying time variable), educational level (no degree, degree, unknown), smoking status (never, former, current, unknown), marital status (married, not married, unknown), diabetes (yes, no, unknown), physical activity (inactive, moderately inactive, moderately active, active, unknown), height (<170, 170–174, 175–179, ≥ 180 cm, unknown), body mass index (<22.5, 22.5–24.9, 25–29.9, ≥ 30 kg/m^2^, unknown), and total energy intake (fifths).

a HR (95% CI) estimated per 100 g/day unit increase in fruit intake.

b *p*‐values for trend were obtained using a pseudo‐continuous variable equal to the median value in each fifth of intake.

c *p* values from test for heterogeneity for the associations of fruit intake with risk of prostate cancer categorized according to prostate tumor grade (low‐intermediate or high) and stage (localized or advanced).

Low‐intermediate grade (Gleason score of <8, or grade coded as well, moderately, or poorly differentiated). High grade (Gleason score of ≥ 8, or grade coded as undifferentiated). Localized stage (TNM staging score of T0‐T2 and N0/Nx and M0, or stage coded in the recruitment center as localized). Advanced stage (T3‐T4 and/or N1‐N3 and/or M1, and/or stage coded in the recruitment center as metastatic).

**Table 5:** Multivariable‐adjusted hazard ratios (95% CI) for prostate cancer per unit increase (per 100 g/day) of vegetable intake in 142,239 men in EPIC (1992–2013)

		Observed intake			Calibrated intake		
	No. of cases	HR (95% CI)^a^	*p*‐trend^b^	*p* for het.^c^	HR (95% CI)^a^	*P*‐trend^b^	*P* for het.^c^
Total vegetables							
Total PCa	7,036	1.01 (0.98–1.03)	0.6		1.01 (0.95–1.07)	0.7	
Grade							
Low‐intermediate	3,757	0.99 (0.95–1.03)	0.5		0.97 (0.89–1.05)	0.5	
High	726	1.08 (1.00–1.17)	0.06	0.05	1.19 (1.00–1.41)	0.06	0.04
Stage							
Localized	2,641	1.02 (0.98–1.07)	0.3		1.05 (0.95–1.16)	0.4	
Advanced	1,389	1.02 (0.96–1.08)	0.6	0.9	1.03 (0.90–1.18)	0.7	0.8
PCa death	936	1.05 (0.98–1.13)	0.2		1.11 (0.95–1.30)	0.2	
Cruciferous vegetables							
Total PCa	6,394	1.06 (0.90–1.23)	0.50		1.08 (0.76–1.54)	0.7	
Grade							
Low‐intermediate	3,281	0.97 (0.77–1.21)	0.8		0.78 (0.46–1.34)	0.4	
High	647	1.26 (0.75–2.09)	0.4	0.3	2.10 (0.58–7.68)	0.3	0.2
Stage							
Localized	2,085	1.03 (0.78–1.36)	0.8		0.90 (0.47–1.70)	0.7	
Advanced	1,303	1.04 (0.72–1.50)	0.8	0.9	1.11 (0.43–2.89)	0.8	0.7
PCa death	882	1.30 (0.86–1.96)	0.2		1.72 (0.65–4.53)	0.3	
Leafy vegetables							
Total PCa	6,394	1.07 (0.91–1.25)	0.4		1.19 (0.86–1.66)	0.3	
Grade							
Low‐intermediate	3,281	0.94 (0.76–1.17)	0.6		0.97 (0.61–1.55)	0.9	
High	647	2.66 (1.54–4.58)	<0.001	<0.001	8.98 (2.79–28.97)	<0.001	<0.001
Stage							
Localized	2,085	1.10 (0.82–1.48)	0.5		1.37 (0.74–2.54)	0.3	
Advanced	1,303	1.19 (0.78–1.79)	0.4	0.8	1.53 (0.66–3.54)	0.3	0.8
PCa death	882	1.26 (0.79–2.00)	0.3		1.95 (0.79–4.83)	0.1	
Fruiting vegetables							
Total PCa	7,036	1.00 (0.95–1.06)	0.9		1.00 (0.92–1.10)	0.9	
Grade							
Low‐intermediate	3,757	1.01 (0.94–1.09)	0.8		1.01 (0.90–1.15)	0.8	
High	726	1.03 (0.86–1.24)	0.7	0.8	1.07 (0.80–1.44)	0.6	0.7
Stage							
Localized	2,641	1.03 (0.94–1.14)	0.5		1.04 (0.89–1.21)	0.6	
Advanced	1,389	1.03 (0.89–1.19)	0.7	0.9	1.06 (0.85–1.33)	0.6	0.8
PCa death	936	1.07 (0.91–1.25)	0.4		1.12 (0.88–1.43)	0.3	
Tomatoes							
Total PCa	7,036	1.05 (0.97–1.15)	0.2		1.09 (0.96–1.24)	0.2	
Grade							
Low‐intermediate	3,757	1.01 (0.90–1.14)	0.8		1.03 (0.86–1.23)	0.7	
High	726	1.10 (0.82–1.48)	0.5	0.6	1.30 (0.84–2.00)	0.2	0.3
Stage							
Localized	2,641	1.00 (0.86–1.16)	0.9		1.03 (0.83–1.28)	0.8	
Advanced	1,389	1.07 (0.86–1.33)	0.6	0.6	1.18 (0.86–1.63)	0.3	0.5
PCa death	936	1.20 (0.93–1.54)	0.2		1.38 (0.97–1.97)	0.07	
Root vegetables							
Total PCa	7,036	1.05 (0.91–1.21)	0.5		1.05 (0.74–1.49)	0.8	
Grade							
Low‐intermediate	3,757	0.95 (0.77–1.17)	0.6		0.76 (0.45–1.27)	0.3	
High	726	1.43 (0.92–2.22)	0.1	0.09	2.10 (0.67–6.54)	0.2	0.1
Stage							
Localized	2,641	1.07 (0.83–1.37)	0.6		0.99 (0.54–1.82)	0.9	
Advanced	1,389	0.89 (0.64–1.23)	0.5	0.4	0.67 (0.29–1.54)	0.3	0.5
PCa death	936	1.07 (0.74–1.55)	0.7		1.27 (0.50–3.22)	0.6	

PCa: prostate cancer.

Cox regression analysis. All models are stratified by center and age at recruitment and adjusted for age (underlying time variable), educational level (no degree, degree, unknown), smoking status (never, former, current, unknown), marital status (married, not married, unknown), diabetes (yes, no, unknown), physical activity (inactive, moderately inactive, moderately active, active, unknown), height (<170, 170–174, 175–179, ≥ 180 cm, unknown), body mass index (<22.5, 22.5–24.9, 25–29.9, ≥ 30 kg/m^2^, unknown), and total energy intake (fifths).

a HR (95% CI) estimated per 100 g/day unit increase in vegetable intake.

b *p*‐values for trend were obtained using a pseudo‐continuous variable equal to the median value in each fifth of intake.

c *p* values from test for heterogeneity for the associations of vegetable intake with risk of prostate cancer categorized according to prostate tumor grade (low‐intermediate or high) and stage (localized or advanced).

Low‐intermediate grade (Gleason score of <8, or grade coded as well, moderately, or poorly differentiated). High grade (Gleason score of ≥ 8, or grade coded as undifferentiated). Localized stage (TNM staging score of T0‐T2 and N0/Nx and M0, or stage coded in the recruitment center as localized). Advanced stage (T3‐T4 and/or N1‐N3 and/or M1, and/or stage coded in the recruitment center as metastatic).

There was no significant heterogeneity for the association between fruit or vegetable intake and total prostate cancer risk when subdivided by age at recruitment (<65 years, ≥65 years), age at diagnosis (<65 years, ≥65 years), by time between recruitment and diagnosis (<5 years, ≥5 years), BMI (<25 kg/m^2^, ≥25 kg/m^2^), and country (Denmark, Germany, Greece, Italy, The Netherlands, Spain, Sweden, UK) (Supporting Information Tables 1 and 2).

## Discussion

In this large European prospective study, a higher intake of total fruit was associated with a small reduction in prostate cancer risk, while vegetable consumption was not related to prostate cancer risk. When consumption was analyzed according to fruit and vegetable subtypes, we found that citrus fruits were weakly associated with a reduced risk of prostate cancer. These associations did not differ by tumor grade or stage, with the exception of leafy vegetables, for which a positive association with high grade prostate cancer was found.

Although in the current large prospective study we observed an inverse association between total fruit consumption and prostate cancer development, the WCRF/AICR meta‐analysis, which included a total of 16 prospective studies with 26,671 cases of prostate cancer, showed a null association between fruit consumption and total prostate cancer risk [Relative Risk per 100 g/day intake 1.00 (95% CI 0.99–1.01)] in 2014.^3^ Only one small prospective study (139 incident prostate cancer cases) on fruit consumption and prostate cancer risk has been published since then, which also showed no association.^22^ If there is an association between fruit consumption and prostate cancer risk it might be due to the high content of vitamins (such as vitamin C) and phytochemicals (such as phenolic compounds and carotenoids), which may have anti‐carcinogenic properties.^10^, ^23^ However, men who have high fruit intake differ in several respects from men with a low fruit intake and therefore residual confounding cannot be excluded. In this study, total vegetable consumption was not associated with overall prostate cancer risk, which is in agreement with WCRF/AICR meta‐analysis.^3^

When fruit and vegetables were divided into subtypes, we found a weak inverse association between citrus fruit and incidence of prostate cancer, which was significant when citrus fruit was introduced continuously per 100 g/day increments. Only one^24^ out of the five^5^, ^8^, ^9^, ^24^, ^25^ studies that have assessed the association between citrus fruit and prostate cancer risk has also found a significant inverse association, and no association between circulating concentrations of the citrus biomarker *β*‐cryptoxanthin and prostate cancer risk was observed in a pooled analysis of 10 prospective studies.^26^ Our non‐significant associations between several vegetable subtypes and risk of prostate cancer are in line with previous prospective studies which have also found no association between overall prostate cancer risk and cruciferous^9^, ^12^, ^27^ or leafy vegetable intake.^8^, ^9^, ^28^, ^29^ Also, although early reports linked frequent consumption of tomatoes, tomato products or lycopene (a carotenoid from tomatoes) with lower risk of overall prostate cancer,^13^, ^25^ our study and the latest meta‐analysis from WCRF/AICR did not support this association,^3^ and nor did findings from a pooled analysis of blood lycopene concentrations and overall prostate cancer risk (although there was statistically significant heterogeneity by stage of disease, and the odds ratios (ORs) for aggressive disease for the highest compared with the lowest fifth of lycopene was 0.65 (95% CI: 0.46, 0.91; *p‐*trend = 0.032).^26^ However, it should be highlighted that not all studies have divided fruit and vegetable intake in the same subtypes.

As far as we are aware, no other large prospective study has examined the association of fruit and vegetable intake with prostate cancer risk separately by both grade and stage of the tumor, and only two studies have analyzed this association with prostate cancer death as the outcome.^7^, ^30^ The latest WCRF/AICR meta‐analysis showed the associations of fruit and vegetable intake with aggressive prostate cancer, a categorization which combined high grade, advanced stage, and prostate cancer mortality.^3^ In the current study we found no association between fruit or vegetable intake and prostate cancer death, or any evidence that any association differed by tumor grade or stage, with the exception of the association of leafy vegetables by tumor grade, with a positive association being limited to high grade prostate cancer only. While leafy vegetables are good sources of folate and circulating folate has been related to high grade prostate cancer,^31^ this association may be a chance finding given the multiple tests. To our knowledge, one previous prospective study has found a positive association between leafy vegetables intake and non‐localized or high grade prostate cancer risk,^9^ but this study did not evaluate associations separately for grade and stage of the disease.

Some strengths and limitations of the present study should be considered. The major strengths of this study include the prospective design, the large number of total prostate cancer cases and prostate cancer deaths and the large amount of grade and stage information, and the reliable identification of prostate cancer cases through cancer registries and/or verified medical records. The Gleason grade was based on data available from biopsies and surgical pathology and there may be some misclassification because of changes in grading over time. The dietary questionnaires in all EPIC centers were validated and dietary intakes were calibrated using measures from a standardized 24‐hr diet recall method, with the aim of correcting for over and under‐estimation of dietary intake.^17^ We were able to look at eight subgroups of fruit and vegetables, although we were not able to look at further subtypes, such as grapes and berries, because median intakes were very low. A limitation of this study was that fruit and vegetable intake was estimated using dietary assessment questionnaires only at baseline, and their consumption may have changed during follow‐up and resulted in exposure misclassification. However, if this is the case, it would have introduced non‐differential misclassification, which tends to bias associations towards the null association. Moreover, as with every observational study, we cannot exclude the possibility of residual confounding by other potential risk factors, such as prostate‐specific antigen (PSA) testing which was not available in our cohort. A recent study in the UK has found that those who consume a higher amount of fruit are more likely to have a PSA test^32^; since those who have had a PSA test are more likely to be diagnosed with prostate cancer, it is unlikely that adjustment for this variable would have changed the associations found. Finally, the increment unit used in our study was 100 g/day in order to provide comparability with previous publications including the WCRF meta‐analysis; however, it should be acknowledged that although this increment is appropriate for major food groups, it is relatively large for some specific food subgroups.

The findings from this large prospective study in European men suggest that higher total fruit consumption may be associated with a small reduction in prostate cancer risk. Some weak evidence of an inverse association between citrus fruit and overall prostate cancer risk, and a positive association between leafy vegetables with high grade prostate cancer was observed. More data are needed from large observational studies with long‐term follow‐up, fruit and vegetable subtypes, and prostate cancer risk by grade and stage of the tumor and prostate cancer mortality before conclusions on risk can be drawn.

## Supporting information

Supporting Information
