# Supplementary material for: Fruit and vegetable intake and prostate cancer risk in the European Prospective Investigation into Cancer and Nutrition (EPIC)
Source: Int J Cancer. 2017 May 15;141(2):287–97. doi: 10.1002/ijc.30741 (PMC5488166; doi:10.1002/ijc.30741)
Supplement: Supplementary file 1 — Supporting Information [file IJC-141-287-s001.docx]

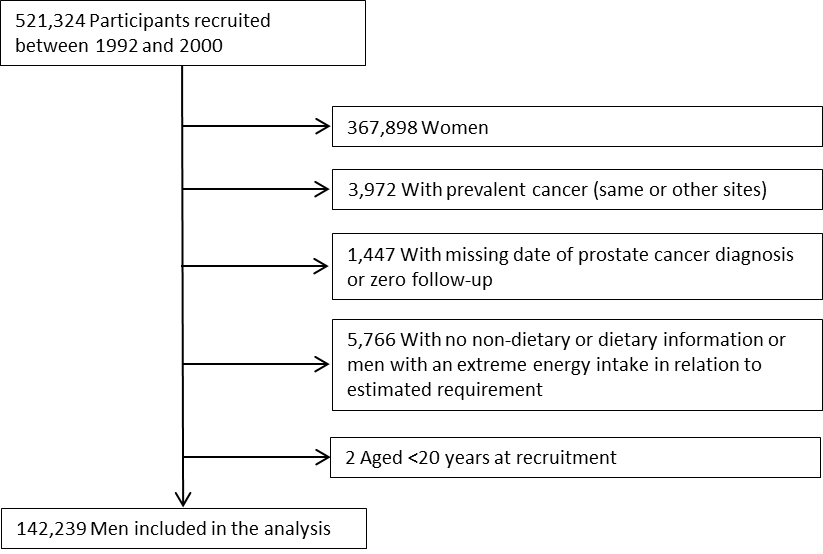


**Supplemental figure 1:** Flow chart describing the selection process among participants of the EPIC prospective study to be included in the present analyses.

| **Supplemental table 1.** Multivariable-adjusted hazard ratios (95 % CI) for prostate cancer in relation to total fruit intake at baseline in 142,239 men in EPIC (1992-2013) by selected characteristics. | | | | | | | |
| --- | --- | --- | --- | --- | --- | --- | --- |
|  |  | **Observed intake** | | | **Calibrated intake** | | |
| **Factor and subset** | **Cases** | **HR (95% CI)^1^** | ***P*-trend^2^** | ***P* for het.^3^** | **HR (95% CI)^1^** | ***P*-trend^2^** | ***P* for het.^3^** |
| Overall | 7036 | 0.97 (0.95 - 0.99) | 0.01 |  | 0.96 (0.94 - 0.99) | 0.006 |  |
| Age at recruitment, y |  |  |  | 0.08 |  |  | 0.09 |
| <65 | 6162 | 0.97 (0.95 - 0.99) | 0.004 |  | 0.96 (0.93 - 0.98) | 0.002 |  |
| ≥65 | 874 | 1.01 (0.95 - 1.07) | 0.8 |  | 1.01 (0.94 - 1.08) | 0.8 |  |
| Age at diagnosis, y |  |  |  | 0.5 |  |  | 0.4 |
| <65 | 2109 | 0.99 (0.95 - 1.03) | 0.6 |  | 0.98 (0.93 - 1.04) | 0.5 |  |
| ≥65 | 4927 | 1.00 (0.98 - 1.03) | 0.8 |  | 1.01 (0.98 - 1.04) | 0.6 |  |
| Time between recruitment and diagnosis, y |  |  |  | 0.6 |  |  | 0.5 |
| < 5 | 1007 | 0.99 (0.93 - 1.04) | 0.7 |  | 0.99 (0.92 - 1.06) | 0.7 |  |
| ≥ 5 | 6029 | 0.97 (0.95 - 0.99) | 0.01 |  | 0.96 (0.94 - 0.99) | 0.008 |  |
| Body mass index, kg/m^2^ |  |  |  | 0.7 |  |  | 0.6 |
| < 25 | 2465 | 0.96 (0.93 - 1.00) | 0.04 |  | 0.95 (0.91 - 1.00) | 0.03 |  |
| ≥ 25 | 4526 | 0.98 (0.96 - 1.00) | 0.1 |  | 0.97 (0.94 - 1.00) | 0.06 |  |
| Country |  |  |  | 0.6 |  |  | 0.7 |
| Denmark | 1885 | 0.97 (0.94 - 1.01) | 0.1 |  | 0.97 (0.92 - 1.01) | 0.2 |  |
| Germany | 833 | 0.93 (0.85 - 1.01) | 0.09 |  | 0.92 (0.83 - 1.01) | 0.09 |  |
| Greece | 97 | 1.01 (0.90 - 1.13) | 0.8 |  | 1.00 (0.78 - 1.29) | 0.9 |  |
| Italy | 479 | 0.99 (0.95 - 1.04) | 0.7 |  | 0.99 (0.90 - 1.09) | 0.9 |  |
| The Netherlands | 215 | 0.98 (0.88 - 1.09) | 0.7 |  | 0.96 (0.80 - 1.14) | 0.6 |  |
| Spain | 666 | 0.98 (0.94 - 1.01) | 0.1 |  | 0.96 (0.92 - 1.01) | 0.08 |  |
| Sweden | 1833 | 0.98 (0.94 - 1.02) | 0.2 |  | 0.95 (0.87 - 1.03) | 0.2 |  |
| United Kingdom | 1028 | 1.01 (0.97 - 1.05) | 0.7 |  | 1.01 (0.96 - 1.07) | 0.7 |  |
| Cox regression analysis. All models are stratified by center and age at recruitment and adjusted for age (underlying time variable), educational level (no degree, degree or unknown), smoking status (never, former, current or unknown), marital status (married, not married, unknown), diabetes (yes, no, unknown), physical activity (inactive, moderately inactive, moderately active, active, unknown), height (<170, 170–174, 175–179, ≥ 180 cm or unknown), body mass index (<22.5, 22.5–24.9, 25–29.9, ≥ 30 kg/m^2^ or unknown), and total energy intake (fifths).  ^1^ HR (95% CI) estimated per 100 grams increase in total fruit consumption.  ^2^ *P*-values for trend were obtained using a pseudo-continuous variable equal to the median value in each fifth of intake.  ^3^ *P*-value from test for heterogeneity for the associations of fruit intake with risk of prostate cancer between subgroups. | | | | | | | |

| **Supplemental table 2.** Multivariable-adjusted hazard ratios (95 % CI) for prostate cancer in relation to total vegetable intake at baseline in 142,239 men in EPIC (1992-2013) by selected characteristics. | | | | | | | |
| --- | --- | --- | --- | --- | --- | --- | --- |
|  |  | **Observed intake** | | | **Calibrated intake** | | |
| **Factor and subset** | **Cases** | **HR (95% CI)**^1^ | ***P*-trend**^2^ | ***P* for het.^3^** | **HR (95% CI)**^1^ | ***P*-trend**^2^ | ***P* for het.^3^** |
| Overall | 7036 | 1.01 (0.98 - 1.03) | 0.6 |  | 1.01 (0.95 - 1.07) | 0.7 |  |
| Age at recruitment, y |  |  |  | 0.2 |  |  | 0.8 |
| <65 | 6162 | 1.00 (0.97 - 1.03) | 0.9 |  | 1.00 (0.94 - 1.07) | 0.9 |  |
| ≥65 | 874 | 1.04 (0.97 - 1.12) | 0.3 |  | 1.09 (0.94 - 1.27) | 0.3 |  |
| Age at diagnosis, y |  |  |  | 0.6 |  |  | 0.6 |
| <65 | 2109 | 1.02 (0.96 - 1.08) | 0.5 |  | 1.04 (0.92 - 1.18) | 0.5 |  |
| ≥65 | 4927 | 1.00 (0.96 - 1.03) | 0.9 |  | 1.01 (0.94 - 1.08) | 0.8 |  |
| Time between recruitment and diagnosis, y |  |  |  | 0.7 |  |  | 0.9 |
| < 5 | 1007 | 1.02 (0.95 - 1.09) | 0.6 |  | 1.03 (0.88 - 1.21) | 0.7 |  |
| ≥ 5 | 6029 | 1.01 (0.98 - 1.04) | 0.7 |  | 1.02 (0.95 - 1.08) | 0.6 |  |
| Body mass index, kg/m^2^ |  |  |  | 0.3 |  |  | 0.6 |
| < 25 | 2465 | 0.99 (0.95 - 1.04) | 0.8 |  | 0.98 (0.89 - 1.08) | 0.7 |  |
| ≥ 25 | 4526 | 1.02 (0.98 - 1.05) | 0.3 |  | 1.03 (0.96 - 1.11) | 0.4 |  |
| Country |  |  |  | 0.9 |  |  | 0.7 |
| Denmark | 1885 | 1.02 (0.97 - 1.08) | 0.4 |  | 1.08 (0.95 - 1.22) | 0.2 |  |
| Germany | 833 | 1.05 (0.93 - 1.19) | 0.4 |  | 1.18 (0.89 - 1.56) | 0.2 |  |
| Greece | 97 | 1.00 (0.89 - 1.14) | 0.9 |  | 0.98 (0.47 - 2.06) | 0.9 |  |
| Italy | 479 | 1.05 (0.95 - 1.16) | 0.3 |  | 1.14 (0.92 - 1.43) | 0.2 |  |
| The Netherlands | 215 | 1.02 (0.78 - 1.33) | 0.9 |  | 0.93 (0.53 - 1.63) | 0.8 |  |
| Spain | 666 | 0.98 (0.93 - 1.03) | 0.3 |  | 0.93 (0.81 - 1.07) | 0.3 |  |
| Sweden | 1833 | 0.98 (0.92 - 1.03) | 0.4 |  | 0.90 (0.78 - 1.05) | 0.2 |  |
| United Kingdom | 1028 | 1.00 (0.95 - 1.05) | 0.9 |  | 1.01 (0.74 - 1.37) | 0.9 |  |
| Cox regression analysis. All models are stratified by center and age at recruitment and adjusted for age (underlying time variable), educational level (no degree, degree, unknown), smoking status (never, former, current, unknown), marital status (married, not married, unknown), diabetes (yes, no, unknown), physical activity (inactive, moderately inactive, moderately active, active, unknown), height (<170, 170–174, 175–179, ≥ 180 cm, unknown), body mass index (<22.5, 22.5–24.9, 25–29.9, ≥ 30 kg/m^2^, unknown), and total energy intake (fifths).  ^1^ HR (95% CI) estimated per 100 grams increase in total vegetable intake.  ^2^ *P*-values for trend were obtained using a pseudo-continuous variable equal to the median value in each fifth of intake.  ^3^ *P*-value from test for heterogeneity for the associations of vegetable intake with risk of prostate cancer between subgroups. | | | | | | | |
